# Leveraging artificial intelligence: Proceeding with caution

**DOI:** 10.4102/jcmsa.v2i1.166

**Published:** 2024-12-18

**Authors:** Soraya Seedat

**Affiliations:** 1 Department of Psychiatry, Faculty of Medicine and Health Sciences, Stellenbosch University, Cape Town, South Africa

Not being particularly tech-literate myself, I have wondered how artificial intelligence (AI) (viz, the systems, machines and software that mimic human intelligence and behaviour) can be practically useful and time saving to medical and dental specialists confronted on a daily basis with the myriad of challenges that demand quick-thinking and innovative solutions. Amid pressing human and financial resource limitations, and in an increasingly complex and interconnected world where work often crosses geographic boundaries, many of us are grappling with *how* and *to what extent* we should let AI into our work lives. Whether we like it or not, AI applications, which include natural language processing (NLP) and machine learning (ML), are potent tools and a disruptive force in healthcare delivery, teaching and training, information-sharing, education of patients, and scientific research and publication practices, including the way we conduct, write up and review research. Many of us agree that AI should complement, not replace, human expertise, fostering a collaborative environment where specialists work in tandem with intelligent systems, machines and software. Many of us would argue that human touch and empathy, provision of a holding space for patients and their families, and appreciation of ethical judgement, emotional salience and cultural nuance, cannot after all be replaced by AI.^1^

With the shortage of specialists and limited patient access to tertiary and specialised health care facilities, particularly in historically underserved provinces and rural regions of the country, AI is bridging service, outreach, and training gaps by enabling remote consultations and diagnostic assistance. Telemedicine, powered by AI algorithms, has for years allowed specialists to remotely assess patients’ conditions and provide expert guidance, irrespective of physical location. This not only extends the reach of medical expertise but also optimises resource allocation. Diagnostic accuracy remains a critical concern for medical specialists. Artificial intelligence-driven diagnostic tools can enhance the accuracy of clinical assessments by analysing vast amounts of data, identifying patterns in the data and providing actionable insights. Machine learning algorithms can help detect anomalies in medical images, such as X-rays and magnetic resonance imaging (MRI) scans, with remarkable precision. This not only speeds up the diagnostic process but also reduces the likelihood of errors, enabling specialists to make more informed decisions.

Treatment personalisation is an essential aspect of present-day healthcare delivery but is often hindered by the complexity of individual patient profiles. Certainly in my discipline (psychiatry), this is the case, where vast heterogeneity in clinical presentation is fascinating and frustrating in equal measure. Generative AI tools that generate content, such as text, images or video, especially natural language processing models, are particularly appealing in the field of psychiatry, considering the centrality of language in the diagnostic and therapeutic process and with several of the core symptoms of psychiatric disorders manifest in spoken language (e.g., pressure of speech in mania, slowed speech in depression and disorganised speech in schizophrenia).^2^ While systematic evaluation of generative AI in psychiatry is fast growing, the vast majority of studies in psychiatry (*n* = 40) that were identified in a systematic review published this month ‘focused on natural language implementations of generative AI, particularly ChatGPT, either by testing its psychiatric knowledge base or evaluating its capabilities as a mental health conversational companion’. Importantly, most studies were pilot or feasibility studies and did not follow clear reporting guidelines (e.g., TRIPOD-LLM) for generative AI.^2^

Big data is the fuel on which AI runs and the ability to link and aggregate datasets and improve healthcare at scale is alluring. Artificial intelligence-driven predictive modelling can analyse patient data, including medical history, genetics, environmental and lifestyle factors, to tailor treatment plans. This level of customisation ensures that patients receive interventions that are optimised for their unique circumstances, potentially improving outcomes and reducing adverse effects. Such a personalised approach empowers medical specialists to make more informed decisions about treatment strategies. Healthcare systems globally are also grappling with rising healthcare costs. Artificial intelligence has the potential to contribute to cost reduction through operational efficiencies and preventive care strategies. Predictive analytics can forecast disease outbreaks, enabling timely interventions that prevent the spread of infections and minimise treatment costs. Additionally, AI-powered chatbots and virtual health assistants can handle routine patient inquiries, freeing up medical specialists to focus on more complex cases. Additionally, by streamlining administrative tasks, AI enables specialists to dedicate more time to patient care.

Another pivotal challenge is the rapid pace of medical research and the overwhelming volume of new information. Medical and dental specialists are tasked with staying up-to-date with the latest developments, which can be overwhelming. Artificial intelligence-powered literature search and analysis tools can efficiently sift through vast amounts of medical literature, extracting relevant information and summarising key findings, thereby reducing the burden on clinicians, researchers, authors editors and reviewers.

While AI holds immense promise, it is crucial that we address the ethical challenges head-on. Central to this are considerations of accountability, responsibility, transparency and respect for patient rights. Patient privacy and data security are paramount; so too is the need for robust protocols on AI use to ensure human oversight of decision-making and to address issues such as ‘function creep’ of the data, commercialisation of personal data and information, and mitigation of unfair biases and discrimination in the data (e.g., on the basis of age, sex, race or geographic representation).^3,4^ Recently, ASSAf and SciELO published research guidelines that specifically cover standards and practices for using AI tools and resources, intended for authors, editors and reviewers.^5^ Authors bear responsibility for their work as authors, and use of AI tools to generate content must be acknowledged and appropriately referenced. This can be done by referencing and citing AI tools and including notes in the methodology or acknowledgements section of a manuscript outlining how AI was used, in line with a specific journal’s guidelines. Editors need to document and disclose any assistance provided by AI tools in the receipt of manuscripts and during evaluation, review and editing. Similarly, peer reviewers need to document use of AI tools in their review reports and be able to vouch for the integrity of the scientific review process. In sum, responsible and ethical deployment of AI should be the cornerstones of all that we do in clinical practice, teaching and training, and research.
